# Prevalence of self-reported asthma among female ice hockey players in the Swedish women's hockey league

**DOI:** 10.3389/fspor.2026.1854629

**Published:** 2026-07-20

**Authors:** Amanda Lahti, Ida Lindman, Emelie Stenman, Anton Grundberg, Kristina Sundquist

**Affiliations:** 1 Center for Primary Health Care Research, Department of Clinical Sciences Malmö, Lund University, Malmö, Sweden; 2 University Clinic Primary Care, Skåne University Hospital, Malmö, Sweden; 3 General Practice/Family Medicine, School of Public Health and Community Medicine, Institute of Medicine, Sahlgrenska Academy, University of Gothenburg, Gothenburg, Sweden; 4 Department of Clinical Sciences, Malmö, Research, Education, Development & Innovation, Primary Health Care, Götaland, Sweden

**Keywords:** allergy, asthma, body checking, female athlete, ice hockey

## Abstract

**Background:**

Asthma is common among athletes, particularly in winter sports, and can affect both health and performance. Ice hockey players are exposed to cold, dry air and high-intensity exertion, all recognized as triggers for asthma symptoms. However, the prevalence of asthma among female ice hockey players remains largely unknown.

**Methods:**

In this cross-sectional study, all 224 players in the 2023–2024 season of the Swedish Women's Hockey League (SDHL) were invited to an online questionnaire that included self-reported asthma, allergy, and medication use.

**Results:**

A total of 154 players (69%) (mean age 23.3, standard deviation 4.4 years) participated. Of these, 26 (17%) had self-reported asthma, and 43 (28%) had a first-degree relative with asthma. Among players with self-reported asthma, 7 (27%) used regular medication, including corticosteroids and short-acting (SABA) and long-acting beta-agonists (LABA). Allergies were self-reported by 44 players (29%). SDHL players had 1.7 times higher odds of self-reported asthma than age-matched females in the Swedish general population (*p* = 0.008).

**Conclusions:**

Female elite ice hockey players reported a higher prevalence of asthma compared with age-matched females in the Swedish general population. These findings highlight the importance of increased awareness, appropriate screening, and continued monitoring of respiratory health in female ice hockey players, while also providing a basis for future longitudinal research as women's ice hockey continues to grow and remains underrepresented in sports medicine research.

## Introduction

Asthma is a disease characterized by airway obstruction, affecting approximately 6%–10% (1, 2). Among athletes, reported asthma prevalence ranges from 10% to 55% depending on sport and competitive level (3, 4), with particularly high rates in winter sports such as skiing and ice hockey (3, 5, 6).

The increased risk of asthma in elite athletes is mainly attributed to the high ventilatory demands of prolonged exercise, where repeated inhalation induces airway dehydration and thermal shifts, triggering vascular leakage, edema, and bronchoconstriction. Recurrent epithelial microtrauma further enhances airway hyperreactivity, promoting asthma development (7–9). Ice hockey players are further exposed to environmental triggers, such as cold and dry indoor air (10), and high carbon monoxide (CO), nitrogen dioxide (NO2), and ultrafine particulate matter (PM_1_) from ice resurfacing machines (11). In addition, asthma could be associated with allergic diseases, highlighting the importance of allergy assessment (12).

In male ice hockey players, asthma prevalence ranges from 14% to 22%, which is markedly higher than in controls (6, 13). However, data on female players are lacking, despite the recent introduction of body checking. Since 2022, the Swedish Women's Hockey League (SDHL) permits body checking, further elevating cardiovascular and ventilatory load in a high-intensity sport already characterized by repeated near-maximal heart rate and ventilation volumes exceeding 100 L·min⁻¹ (14).

Moreover, previous research has suggested that asthma may be more common among female athletes than male athletes (15, 16), even though findings remain inconsistent across sports and study populations (17, 18). Despite this, female athletes and female ice hockey players remain markedly understudied in sports medicine research (19). As women's ice hockey continues to grow and evolve, sport- and sex-specific studies are needed to better understand potential respiratory health risks in this population (20).

The aim of this study was to determine the prevalence of self-reported asthma and allergy among female SDHL players and compare it with the general Swedish female population.

## Methods

The present study is based on baseline data from the *Women's Ice Hockey Injury Study*, a research project with the broader aim of examining injuries and general health among players within the SDHL, which in 2022 was the first women's ice hockey league worldwide to allow body checking (21, 22). The league consists of 10 teams, each with approximately 20–25 players.

An invitation email was distributed to all 224 athletes registered in the SDHL during the 2023/2024 season. Participation was voluntary, with the option to discontinue at any time. E-mail addresses to the players were obtained from representatives from the SDHL and the clubs' medical teams, who were involved in supporting communication with clubs. However, they did not have access to the data nor participated in the analysis of this study.

The e-mail contained written information about study aims, ethical considerations of the research project as well as a consent form that all players that accepted participation filled in electronically. Of all 224 players invited to participate, 154 players (69%) consented to participate, including 21 players (14%) below the age of 18 (range 16–37 years). The players represented all teams and playing positions in SDHL. A prior dropout analysis reported no significant age or body height differences between responders and non-responders (22).

Directly after consenting to participate, the players were redirected to an online health questionnaire survey, available in both Swedish and English, which contained a wide range of questions about their demographics, socioeconomics, anthropometrics, health, and playing position. The questionnaire was administered between December 2023 and April 2024, corresponding to the mid-to-late competitive season.

The questionnaire also included items on previous and current diseases, specifically self-reported asthma and allergies, as well as family history of these conditions. No objective diagnostic measures, such as spirometry or bronchial provocation tests, were performed. Accordingly, asthma and allergy were defined as self-reported conditions. Additional questions assessed the use of asthma medication. The following questions were analyzed in the present study:

1. Do you have, or have you previously had, asthma?
2. Do you have, or have you previously had, an allergy?
3. Do you take any medications regularly? (yes, no)
  1.1 State which medicines you use and the strength and dosage of the medicine
4. Do any of your parents or siblings have any of the following conditions or diseases?—Asthma (yes, no, do not know)

Accordingly, self-reported asthma refers to a lifetime diagnosis, including both current and previous asthma. Question 3 referred to all medications and asked participants to report the medication name, dose, and strength (i.e., concentration, such as mg or µg per dose). Based on the responses to question 3, reported medications were classified as asthma medications if they were recognized as such. Classification of the reported medications was performed by a general practitioner and first author of this study (AL) with no clinical responsibility for the players. Because asthma was assessed solely by self-report, misclassification cannot be excluded, potentially leading to both under- and overestimation of the true prevalence.

Smoking and snus (a Swedish moist, smokeless tobacco product placed under the upper lip) use were assessed by the questions “*Do you smoke?*” and “*Do you use snus?*”, respectively, with the response options *Yes/No/Sometimes.* Smoking was defined as answering “*Yes*” or “*Sometimes*.” Alcohol use was assessed by the question “*How often do you drink alcohol*” with response options “*never*”, “*<1 standard drink/week*”, “*1*–*2 standard drinks/week*”, “*3*–*9 standard drinks/week*” and “*more than 9 standard drinks per week*”.

Players also reported the number of years they played ice hockey at an elite level and their age when they began intensive training aimed at performance enhancement. Further, they provided information on country of birth, their own highest educational level (*compulsory school, upper secondary, university courses, or university degree*), and the highest educational level of their mother and father, respectively. Because some players were still of school age and might not yet have completed their education, parental educational level was used in the analyses to assess socioeconomic conditions. This variable was selected since asthma prevalence has previously been shown to be associated with social determinants, including educational level (23).

The questionnaire was not formally validated; however, it was pilot-tested in approximately ten age-matched female athletes to ensure clarity and comprehensibility of the questions prior to distribution to the ice hockey players.

Written informed consent was obtained from all participants in the study. The study complies with the Declaration of Helsinki, received approval from the Swedish Ethical Review Authority (Dnr 2023-06114-01), and is registered at ClinicalTrials.gov.

### Statistical methods

Statistical analyses were performed using R version 4.5.3 (R Core Team, 2026). Descriptive data are presented as mean values with standard deviations (SD) for continuous variables and as numbers and percentages for categorical variables. Comparisons of proportions between the reference population was conducted using Chi-square (*χ*^2^) tests. The reference population represented the general population of Swedish women in the corresponding age (1). These reference datasets were used to provide comparative baselines for the distribution of relevant categorical variables. Odds ratios (OR) with 95% confidence intervals (CI) were calculated for key estimates where appropriate. No formal sample size or power calculation was performed, as the study aimed to include all registered players in the SDHL during the 2023–2024 season. Missing values of self-reported asthma were treated as not having asthma to keep the estimates and *p*-values conservative. To assess any bias introduced by handling missing asthma status this way, we performed a sensitivity analysis as a complete case analysis excluding missing data. Statistical significance was defined as a two-tailed *p*-value <0.05.

## Results

A total of 154 players, with mean age 23.3 (SD = 4.4) years, completed the survey. Descriptive characteristics are presented in Table 1. All ten teams were represented in the study. A total of 85 (55%) were forwards, 52 (34%) were defensemen, and 17 (11%) were goalkeepers. Players started intensive training to enhance performance at a mean age of 13.0 (2.7) years and had been competing at the elite level for 5.2 (4.3) years on average.

**Table 1:** Characteristics of 154 women playing in the Swedish women's hockey league (SDHL) during the 2023–2024 season.

**Characteristic**	**Mean (SD) or *n* (%)** ^a^
Age (years)	23.3 (4.4)
Height (cm)	169.1 (5.5)
Weight (kg)	68.4 (6.8)
BMI (kg/m²)	23.9 (1.9)
Playing position	
Forward	85 (55%)
Defenseman	52 (34%)
Goalkeeper	17 (11%)
**Smoking**	
Yes	0 (0%)
No	152 (99%)
Missing	2 (1%)
**Using snus**	
Yes	22 (14%)
Sometimes	7 (5%)
No	123 (80%)
Missing	2 (1%)
**Alcohol (drinking frequency)**	
Never	46 (30%)
<1 standard drink/week	96 (62%)
1–2 standard drinks/week	10 (6)
≥3 standard drinks/week	0 (0%)
Missing	2 (1%)
**Mother's highest educational level**	
Primary school	2 (1%)
Upper secondary	37 (24%)
Post-secondary	27 (18%)
University/college	72 (47%)
Don't know	14 (9%)
Missing	2 (1%)
**Father's highest educational level**	
Primary school	9 (6%)
Upper secondary	51 (33%)
Post-secondary	22 (14%)
University/college	56 (36%)
Don't know	14 (9%)
Missing	2 (1%)
**Player's country of birth**	
Sweden	85 (55%)
Other Nordic countries (Norway, Finland, Denmark, Iceland)	27 (18%)
Other European countries	24 (16%)
North America (USA, Canada)	12 (8%)
Other countries (e.g., Japan, Russia)	3 (2%)
Missing	3 (2%)
Age at start of intensive training for performance	13.0 (2.7)
Number of years as an elite ice hockey player (years)	5.2 (4.3)

a Continuous variables are presented as mean (SD) and categorical as *n* (%).

None of the players reported smoking, while 29 (19%) used snus daily or occasionally. No player consumed ≥3 standard drinks per week. Mothers and fathers hold a university or college degree in 47% and 36% of cases, respectively. Most players were born in Sweden (55%), followed by other Nordic (18%) and European (16%) countries.

A previous drop out analysis found no differences in age, height, weight or BMI between players who participated in the study and those who did not, neither compared to the non-checking north American league PWHL (22).

Of all players, 26 (17%) had self-reported asthma and 44 (29%) reported having allergies. A total of 17 (11%) reported having both self-reported asthma and allergies. Of those with self-reported asthma, 16 (62%) had at least one family member with asthma and among all players, 43 (28%) had at least one family member with asthma. Among players with self-reported asthma, 7 (27%) used medications regularly, including corticosteroids and short-acting (SABA) and long-acting beta-agonists (LABA) (Table 2).

**Table 2:** Prevalence of self-reported asthma, allergies, use of asthma medication and family history in 154 women playing in the Swedish women's hockey league (SDHL) during the 2023–2024 season. Data is presented as *n* (%).

**Answer**	**Asthma**	**Allergy**	**Regular asthma medication use** ^a^	**Parent or sibling with asthma**	**Parent or sibling with allergy**
Yes	26 (17)	44 (29)	7 (5)	43 (28)	81 (53)
No	122 (79)	104 (68)	147 (95)	99 (64)	61 (40)
Do not know	N/A	N/A	N/A	6 (4)	6 (4)
Missing	6 (4)	6 (4)	0 (0)	6 (4)	6 (4)

a Asthma medication use includes both corticosteroids and/or bronchodilators (SABA/LABA).

Compared with Swedish females aged 16–25 years in the general population, in whom asthma was also self-reported, female ice hockey players had 1.7 higher odds (*p* = 0.01) of self-reported asthma (Table 3). The sensitivity analysis showed similar results (Supplementary Table S1).

**Table 3:** Prevalence of self-reported asthma among women playing in the Swedish women's hockey league (SDHL) during the 2023–2024 season compared with the general population.

**Group**	**Asthma,**	**No Asthma,**	**Total,**	**OR (95% CI)**	***p*-value**
	***n* (%)**	***n* (%)**	***n* (%)**		
Female Elite ice hockey players in SDHL	26 (17%)	128 (83%)	154 (100%)	1.7^a^ (1.1–2.6)	0.01^a^
General female Swedish population aged 16–25 years^a^	10.6%	89.4%	100%	Ref ^a^	

a Reference data from a population-based study of self-reported asthma (*n* = 24 534); exact number of participants for the 16–25 age group was not available (1).

## Discussion

This is the first study to report the prevalence of self-reported asthma among female ice hockey players competing in a league permitting body checking. In the present cohort, 17% of the players reported self-reported asthma, a prevalence that was higher than that observed in an age-matched Swedish general population, also using a self-reported definition of asthma. These findings are consistent with previous reports of a higher occurrence of asthma among athletes, particularly in winter sports with high ventilatory demands and exposure to cold, dry air and air pollution (1–3).

The present study provides a unique league-wide baseline that enables future longitudinal analyses of respiratory health in a rapidly evolving sport. However, they should be interpreted with caution, as asthma was assessed by self-report and environmental and training-related factors were not measured. In elite ice hockey players, respiratory symptoms may reflect a spectrum of underlying conditions rather than asthma alone. Exercise- and cold-induced bronchoconstriction is common in athletes exposed to high ventilatory demands and cold, dry air and may occur independently of an asthma diagnosis. Consequently, self-reported asthma may also represent exercise- and cold-induced bronchoconstriction.

In ice hockey, playing positions differ in physiological demands, with forwards and defensemen engaging in repeated high-intensity, intermittent skating requiring both anaerobic and aerobic capacity, whereas goalkeepers perform more static, reactive movements with lower overall metabolic load. In addition, time on ice varies by position, with skaters typically accumulating greater total playing time than goalkeepers. These differences in workload may influence respiratory stress and could potentially contribute to variation in asthma prevalence across positions. However, the number of players within each positional subgroup in the present study was limited, precluding meaningful subgroup analyses. Future studies in larger cohorts may therefore benefit from exploring potential differences in asthma prevalence across playing positions.

Yet, the self-reported asthma prevalence observed in the present study appears to be similar to that reported in a previous study using objective spirometry testing (24). However, the cited study is more than two decades old and included only one-third as many participants as the present study. The present study therefore provides updated prevalence estimates in a contemporary cohort of female elite ice hockey players, thereby strengthening the evidence with larger-scale data in a previously understudied population.

Competition in women's ice hockey has likely intensified as participation in the sport has grown rapidly in recent years, underscoring the need to update the evidence and continue monitoring respiratory health among female ice hockey players (20). So far, the prevalence of self-reported asthma among SDHL players appears similar to that reported 20 years ago in female U.S. national team players, where body checking was not permitted (24). Whether changes in game intensity, combined with the rapid growth of women's ice hockey, may influence self-reported asthma prevalence in this population, remains uncertain and should be examined in future studies.

In addition to physical training, several lifestyle and socioeconomic factors have been associated with asthma. For example, smokers and snus users have been shown to have a higher prevalence of asthma (23). While the evidence regarding association between alcohol consumption and asthma remains mixed (25, 26).

Asthma is also more common among individuals with obesity, physical inactivity, and lower educational attainment (23). In a large population-based study including over 10,000 Swedish female citizens aged 18–49 years, the prevalence of daily smoking, daily snus use, high-risk drinking of alcohol, obesity, physical inactivity, and low educational level (defined as compulsory education only) were 6%, 12%, 16%, 16%, 31%, and 15%, respectively (23).

In contrast, the study population of female elite ice hockey players represents a considerably healthier group, with substantially lower levels of these established risk factors. It can also be assumed that all players in the present study achieved more than 1­­50 min of physical activity per week, compared with only 69% in the general population of female citizens aged 18–49 years (23). Moreover, the players had a mean BMI of 23.9 (SD 1.9), indicating a normal weight range with no evidence of obesity at the group level.

Nevertheless, the prevalence of self-reported asthma was higher (17%) compared with the general population (9% among women and 7% among men), supporting the rationale presented in the introduction that sport-specific factors, such as high ventilatory demands and environmental exposures, may play a more important role than traditional lifestyle-related risk factors in this population. However, this interpretation should be made with caution, as asthma was assessed by self-report and may partly reflect exercise- and cold-induced bronchoconstriction rather than clinically confirmed asthma, and environmental and training-related factors were not directly measured.

The prevalence of asthma also varies substantially across countries and regions, with higher rates generally reported in Western Europe, North America, Australia, and New Zealand, and lower rates in parts of Asia, Africa, and Eastern Europe (27). These differences may reflect environmental, genetic, and socioeconomic factors, as well as variations in healthcare access and diagnostic practices. Asthma prevalence has also been shown to differ by country of birth, with individuals born outside Europe and North America often exhibiting lower rates, suggesting that early-life exposures and genetic background may influence disease susceptibility (28, 29). In this study, most participants were born in Europe or North America, which may contribute to the relatively high asthma prevalence.

Previous research has reported a higher prevalence of asthma among female athletes compared with their male counterparts (16, 30). A higher prevalence among females has been reported in the general non-athletic population as well (1). A previous study found that 5 of 26 (19%) male ice hockey players had clinically diagnosed asthma following screening with objective pulmonary function testing, including spirometry (31). This prevalence appears comparable to our findings; however, future studies including both male and female players, using objective diagnostic methods and larger sample sizes than those available in the reference study, are needed to allow for more reliable comparisons between male and female elite ice hockey players.

Only about one in four of the players with self-reported asthma in the present study reported regular use of asthma medications. This may highlight a well-described challenge in athlete populations: both under- and overtreatment occur, and asthma symptoms can be easily masked by athletes' high cardio-pulmonary capacity. Under-treatment is of particular concern, since uncontrolled asthma not only reduces performance yet also potentially causing irreversible structural airway changes if left untreated (32). Conversely, overtreatment has historically raised suspicions of performance enhancement, although scientific evidence does not support a true ergogenic effect of inhaled asthma medications when used in recommended doses (33–35).

Structural barriers may also play a role, as access to team physicians, medical services, and diagnostic evaluation may be more limited in women's sports compared with men's leagues (36). This could partly explain why several players report having an asthma diagnosis while relatively few report regular use of medication (36). Considering the relatively high prevalence of self-reported asthma observed, and the risk of irreversible airway changes if asthma remains untreated, it is crucial that elite sports environments are equipped with specialized medical expertise. Such expertise should be capable of detecting, managing, and monitoring asthma in athletes, with a thorough understanding of sport-specific demands and the unique physiology of elite competitors.

The present study is part of a larger ongoing research project monitoring injuries and health among elite women's ice hockey players, enabling longitudinal follow-up of this population. As women's ice hockey evolves and the physical intensity of the women's game increases, particularly following the introduction of body checking, the physiological demands on players, including ventilatory load, may also increase. This may influence respiratory health and potentially the prevalence of asthma over time. The findings of the present study therefore provide important baseline data for monitoring how the sport develops and for identifying potential respiratory health risks associated with an increasingly physical style of play.

Future research should aim to objectively assess the prevalence of physician-diagnosed asthma among female elite ice hockey players and include provocation tests, such as exercise challenges or eucapnic voluntary hyperventilation (37), to determine how common the condition truly is in this population. Additionally, studies should examine asthma symptoms longitudinally as well as their impact on performance and treatment adherence.

## Strengths and limitations

Study strengths include the high response rate (69%) and the larger number of participants compared with previous studies (24, 31). The study also contributes to the limited body of research on female athletes in a historically male-dominated sport and in a field where sex inequalities in sports medicine research persist (19). Another strength is that players from all ten teams and all playing positions within the league were included. In addition, a previous dropout analysis from this cohort showed no anthropometric differences between participants and non-participants, nor compared with players in the, at that time, non-checking Professional Women's Hockey League (PWHL), supporting the representativeness of the study population (22). Extensive data collection covering a wide range of health-related variables allows for a comprehensive characterization of the players and enables analyses considering other relevant background factors such as socioeconomic status and comorbidities, which should be regarded as an additional strength of the study. A key strength is also the homogeneity of the cohort, with almost all participants born within Europe or North America. This minimizes confounding from geographical or ethnic variation and increases the likelihood that observed asthma differences reflect factors related to elite ice hockey participation rather than population diversity.

However, several limitations should also be acknowledged. First, the reliance on self-reported asthma diagnoses may lead to misclassification, potentially under- or overestimating the true prevalence. In addition, the lack of objective diagnostic testing limits the ability to distinguish asthma from exercise- or cold-induced bronchoconstriction, conditions that are highly prevalent among winter sport athletes. Second, the cross-sectional design precludes conclusions about causality or temporal relationships between sport participation and asthma development. Third, comparison with historical population data may be affected by temporal changes in asthma prevalence, diagnostic criteria, and reporting practices (1, 12). Potential confounders such as environmental exposures, training intensity, and atopic status were not controlled for since we did not have access to such data, which could influence the observed ORs when comparing with the referenced study populations. Even more, no priori power calculation was conducted, and the study may be underpowered to detect small differences in comparisons with external reference populations. Non-significant findings should therefore be interpreted cautiously. Finally, allergies in this study were not limited to rhino conjunctivitis or pollen allergies but rather included all forms of allergy (e.g., contact, drug, and food allergies), which may have led to an overestimation of the allergy prevalence. Future longitudinal studies incorporating objective diagnostics, environmental monitoring, and repeated assessments are needed to better understand incidence, progression, and management of asthma in elite female ice hockey players.

## Conclusion

This study demonstrates that Swedish female elite ice hockey players have a substantially higher prevalence of asthma compared with the general population. The findings underscore the importance of early detection, accurate diagnosis, and individualized management of asthma to prevent under-treatment and potential long-term airway remodeling. Regular respiratory health monitoring, access to specialized medical expertise, and adherence to evidence-based treatment strategies are essential to safeguard both health and athletic performance in this high-risk population. In addition, the findings from the present study should be interpreted as descriptive of self-reported respiratory disease rather than true asthma prevalence, and causal or mechanistic conclusions cannot be drawn. Despite these limitations, the study provides valuable league-wide baseline data on self-reported respiratory health in elite female ice hockey players.

## Data Availability

The raw data supporting the conclusions of this article will be made available by the authors, without undue reservation.
