# Supplementary material for: Prevalence of self-reported asthma among female ice hockey players in the Swedish women's hockey league
Source: Front Sports Act Living. 2026 Jul 20;8:1854629. doi: 10.3389/fspor.2026.1854629 (PMC13429784; doi:10.3389/fspor.2026.1854629)
Supplement: Supplementary file 1 [file Datasheet1.pdf]

Supplementary

Table S1. Prevalence of self-reported asthma among 154 women playing in the Swedish Women's Hockey League (SDHL) during the 2023–2024 season and reference data from the general population*. Sensitivity analysis – participants with missing asthma status excluded.

Group	Asthma, n (%)	No Asthma, n (%)	Total, n (%)	OR (95% CI)	p-value
**Female Elite ice hockey players body checking league SDHL**	26 (18%)	122 (82%)	148 (100%)	1.8 (1.2 – 2.8)	0.01
**General female Swedish population aged 16-25 years***	10.6%	89.4%	100%	Ref	

* Reference data from a population-based study of self-reported asthma (n=24 534); exact number for the 16–25 age group was not available (1).
